# Histone Acetylation and Methylation Underlie Oligodendroglial and Myelin Susceptibility in Schizophrenia

**DOI:** 10.3389/fncel.2022.823708

**Published:** 2022-03-10

**Authors:** Mei Li, Lan Xiao, Xianjun Chen

**Affiliations:** ^1^Department of Physiology, Research Center of Neuroscience, College of Basic Medical Science, Chongqing Medical University, Chongqing, China; ^2^Laboratory of Human Physiology, Lab Teaching and Management Center, Chongqing Medical University, Chongqing, China; ^3^Department of Histology and Embryology, Institute of Brain and Intelligence, Army Medical University (Third Military Medical University), Chongqing, China

**Keywords:** oligodendrocyte, myelin, histone acetylation, histone methylation, epigenetic, schizophrenia, psychiatric disorder

## Abstract

Schizophrenia is a complex neuropsychiatric disorder affected by both genetic and epigenetic factors. Except for neuronal dysfunction, oligodendroglial abnormalities also contribute to the disease pathogenesis, characterized by a robust dysregulation of oligodendrocyte and myelin related genes. Accumulating evidence shows that histone modifications play important roles in transcriptional regulation of the genes crucial for oligodendrocyte differentiation and myelination. Specifically, the histone acetylation and methylation were two well-recognized histone modification abnormalities in the schizophrenic brains. In this mini-review, we will describe the dynamic changes of histone acetylation and methylation in schizophrenia, which may coordinate and induce deleterious epigenetic memory in oligodendroglial cells, and further lead to oligodendrocyte and myelin deficits. Precise modulation of histone modification status in oligodendroglial cells needs to secure the balance of epigenetic marks, which may revise the therapeutic strategy for the white matter etiology of neuropsychiatric disorders.

## Introduction

Schizophrenia is a severe and heterogeneous psychiatric disorder that affects about one percent of the world’s population (Jauhar et al., 2022). Notably, the critical period for oligodendrocyte (OL) and myelin development coincides with the peak onset of schizophrenia, which is concentrated in a very narrow time window range from adolescence to young adulthood (Lee et al., 2014; Kwon et al., 2020). In fact, accumulating evidence shows that the structure and function of myelinating OLs are impaired in major neuropsychiatric diseases, including schizophrenia, depression and bipolar disorder (Martins-de-Souza, 2010; Edgar and Sibille, 2012; Dietz et al., 2020). In schizophrenia, even before disease onset, reduced myelin integrity occurs in the frontal areas and this advances to more caudal and posterior regions in further stages of the disorder (Friedman et al., 2008; Liu et al., 2014), indicating that OLs and myelin dysfunction could contribute to the disease etiology, rather than simply accompanied abnormalities (Walterfang et al., 2009). More importantly, current evidence shows an extensive transcriptome alteration in OLs from multiple brain regions of schizophrenic patients, including multiple genes crucial for oligodendroglial development and myelination (Martins-de-Souza, 2010; Horváth et al., 2011), which implies a global epigenetic dysfunction in OLs.

Apart from the genetic architecture, environmental risk factors, including biological and psychosocial ones, could contribute to the mental disease onset *via* epigenetic mechanisms (Sullivan et al., 2012; Owen et al., 2016; Famitafreshi and Karimian, 2019). Environmental risk factors identified so far have implicated prenatal, perinatal and adolescent events (Insel, 2010). For example, social isolation is a kind of stress, which is tightly related to mental illness characterized by reduced sociability (Liu et al., 2012; Makinodan et al., 2012). Both maternal deprivation at infancy and long-term social isolation at adulthood have a profound impact on a variety of behaviors, such as defensive behavior, sociability and cognitive function (Popa et al., 2020; Wang et al., 2020). Strikingly, these environmental risk factors will irreversibly change the composition of oligodendroglial lineage cells (Teissier et al., 2020; Wang et al., 2021), down-regulate myelin related genes, produce OLs with simpler morphology (Makinodan et al., 2012), and impair myelinogenesis in the prefrontal cortex of postnatal and adult mice (Liu et al., 2012; Makinodan et al., 2012; Birey et al., 2017; Lehmann et al., 2017).

Oligodendroglial development is a highly dynamic process that continues into adulthood (Liu et al., 2016), which is secured by the interactions between transcription factors and chromatin modifications (Zuchero and Barres, 2013; Emery and Lu, 2015; Koreman et al., 2018a). Operationally, chromatin modifications function either by disrupting chromatin contacts or by affecting the recruitment of non-histone proteins to chromatin (Kouzarides, 2007). In this regard, histone modifications can affect the ordered chromatin structure, orchestrating the recruitment of enzyme complexes to manipulate DNA (Kouzarides, 2007). In OL, histone modifications play an important role in the regulation of oligodendroglial development (Koreman et al., 2018a). Among various histone modifications, recent study found that histone acetylation and methylation occur most frequently in schizophrenia (Thomas, 2017a), which may lead to lasting changes in gene expression and have therefore been implicated in OL abnormalities that characterize the white matter etiology.

Based on the emerging evidence of histone acetylation and methylation alterations in OL under environmental risk stimuli, we briefly discuss the following issues here: (1) Are there any stage-specific mechanisms for the histone acetylation/methylation during oligodendroglial development? (2) How do these histone modifications negotiate and affect the oligodendroglial development and function? (3) Whether environmental risk stimuli could induce adverse epigenetic memory in OLs and increase the cellular susceptibility?

### Transient Histone Acetylation May Preferentially Affect the Early Differentiation of Oligodendrocyte Precursor Cells

Acetylation of nucleosomal histones modified by histone deacetylases (HDACs) and histone acetyltransferases (HATs) has been identified as epigenetic mechanisms in the regulation of oligodendroglial development (Emery and Lu, 2015). Histone acetylation adds acetyl groups on specific lysine residues by HATs. Histone deacetylation is the removal of acetyl groups from lysine residues mediated by HDACs (Shen et al., 2005). Both HATs and HDACs can be recruited to their binding sites that form transcriptional regulator complexes to modulate gene expression (Tsai and Casaccia, 2019).

So far little has been known about the function of HATs in oligodendroglial development, however, HDACs are well-studied in different aspects of oligodendroglial development. Generally, HDACs function to make the chromatin more compact, thereby blocking the transcriptional processes of target genes (Bannister and Kouzarides, 2011). Notably, HDACs are predominantly expressed in the early oligodendrocyte precursor cell (OPC) stages, as compared to other stages of oligodendroglial lineage cells (Egawa et al., 2019), and are crucial for the early differentiation of OPCs to mature OLs (Conway et al., 2012). Besides of histone deacetylation, HDACs could interact with some transcriptional regulators to indirectly restrict the expression of OPC differentiation inhibitors, thereby facilitating OPC maturation (Ye et al., 2009). Recent study found that blocking HDAC activity impairs the OPC early differentiation, but does not affect myelin gene expression after initiating myelination (Shen et al., 2005). Therefore, global histone acetylation could be the mechanism responsible for the timing of early oligodendroglial development.

Importantly, some studies have found that histone acetylation regulators (e.g., HDACs) were dysregulated in the brain of patients with mental diseases (Nestler et al., 2016). As the effect of HDACs activity seems to be temporally specific at the early phase of OPC development, the dysregulation of HDACs may exhibit diverse roles on oligodendroglial development based on a cellular context and/or stage-specific context. In this way, the dysregulation of HDACs may be detrimental under certain circumstances but beneficial in others. When overall histone acetylation was changed in OLs, the expression of multiple schizophrenia risk factors was altered (Conway et al., 2012; Zhang et al., 2016). However, the locus-specific changes of histone acetylation at a particular gene have not been precisely dissected. Therefore, it is rational to deduce that histone acetylation alterations may preferentially impair OPCs differentiation at the early stage.

### Stable Histone Methylation May Affect Oligodendroglial Development in a Multidirectional Manner

Histone (de)acetylation is transient and reversible in oligodendroglial lineage cells (Emery and Lu, 2015), so acetylation itself is unlikely to be sufficient for OLs differentiation, neither in normal developmental nor in psychiatric situations. Studies have found that the stable inhibitory histone methylation contributes to the differentiation of OPCs into mature OLs (Liu et al., 2015). The enzymatic methylation of histones is performed by lysine methyltransferases (KMTs) and protein arginine methyltransferases (PRMTs) (Guccione and Richard, 2019). Histone methylation can involve the transfer of up to three methyl groups, thus resulting in mono-, di-, or tri-methylated lysine, respectively, and in mono- or di- methylated arginine. In addition, the removal of methyl moieties at these residues are carried out by histone lysine/arginine demethylases (Chang et al., 2007; Jambhekar et al., 2017). Generally, histone methylations exhibit at promoters, insulators, enhancers, and transcribed regions of target genes. Specifically, the monomethylations of H3K27, H3K9, and H3K79 are associated with transcriptionally active promoters, whereas trimethylations of H3K27, H3K9, and H3K79 are linked to transcriptional repression and heterochromatin formation (Boggs et al., 2002; Barski et al., 2007). H3K4 tri-methylation (H3K4me3) is a hallmark of transcriptional start sites and is generally followed by H3K4me2 and H3K4me1 along the coding regions (Barski et al., 2007; Zhang et al., 2009). The various functions of H3K4 depend on four methylation levels including unmethylated, mono-, di- or trimethylated (Shi et al., 2006; Wysocka et al., 2006; Lan et al., 2007). Therefore, as histone methylations occupied different genomic positions or the same position but in different states, they may selectively recruit various chromatin-associated proteins and differently regulate gene expression, and thereafter exhibit different roles at various stages of oligodendroglial differentiation (Liu et al., 2015; Koreman et al., 2018a).

Histone methylation has been shown to be altered in a variety of psychiatric disorders. Huang et al. (2007) found decreased levels of the open chromatin mark, H3K4 tri-methylation, and elevated levels of the repressive mark, H3K27 trimethylation, in post-mortem prefrontal cortex from subjects with schizophrenia. More recently, an increase in global cortical H3K9 di-methylation levels was reported in a schizophrenia cohort in the parietal cortex associated with increases of two of the enzymes that catalyze its formation, GLP and SETDB1 (Chase et al., 2013). In a separate study, Chase et al. (2015) found that men with schizophrenia expressed higher levels of H3K9 di-methylation in conjunction with higher expression levels of G9α, SETDB1 methyltransferase. Moreover, together with Liu et al., we reported that social deprivation, a well-defined environmental risk factor of schizophrenia, could affect H3K9 tri-methylation status in OLs, which could be responsible for OLs dysfunction and myelin deficits in the prefrontal cortex (Liu et al., 2012; Chen et al., 2019). Besides, genetic variants in the gene encoding histone lysine demethylases (e.g., KDM4C) were found to contribute to schizophrenia susceptibility (Kato et al., 2020). The altered H3K4me3 mark regulates growth, metabolism and energetics in OLs, while H3K9me3 is associated with transcriptional repression in developing OLs (Singhal et al., 2017). Therefore, these findings raise a possibility that aberrant histone methylation could affect OLs function when it occurs at risk genes in OLs. Next, more work is needed to determine the causality and possible targets of histone methylation during the disease pathogenesis.

### The Co-ordination of Histone Acetylation and Methylation May Increase the Complexity of Epigenetic Marks in Oligodendrocyte

**FIGURE 1:**
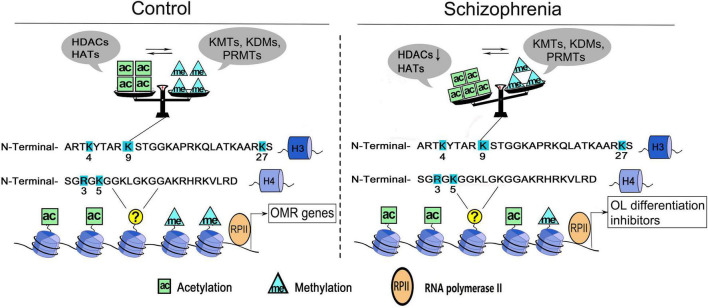
A working model of histone acetylation and methylation underlying OLs dysfunction in schizophrenia. Histone acetylation and methylation at the promoters governing the expression of oligodendrocyte/myelin-related (OMR) genes. Multiple sites of H3 and H4 are dynamically regulated by histone acetyltransferases/deacetylases (e.g., HATs, HDACs) and/or histone methyltransferases/demethylases (e.g., KMTs, KDMs, and PRMTs). The cross-talk between two classes of enzymes secures the balance of epigenetic marks in OLs. In schizophrenia, the dysregulation of enzymes could change the balance/priority of histone acetylation and methylation at some key sites (e.g., H3K9), thus induce the reactivation of OL differentiation inhibitors and increase the cellular susceptibility. ac, histone acetylation; me, histone methylation; H3, histone 3; H4, histone 4.

Histone acetylation and methylation occur at different regions of genes, as the former occurs predominantly at the beginning of genes, whereas the latter occurs throughout the transcribed regions (Pokholok et al., 2005), therefore, they may coordinate to affect the transcriptional activity of target genes. For example, H3K4me3 is often found together with acetylation of many other residues (e.g., K9, K14, K18, K23, and K27) on the same H3 molecules (Zhang et al., 2004; Jiang et al., 2007; Nightingale et al., 2007). H3K4me3 could directly bind to Yng1, a subunit of the NuA3 histone acetyltransferase complex that modifies H3K14, which couples the acetylation and methylation of histone 3 on various residues (Taverna et al., 2006). In OLs, the key lysine residue on H3 is deacetylated at the genetic loci of myelin gene transcription inhibitor during oligodendroglial differentiation, followed by the decrease of repressive H3K27me3 marker and the increase of activated H3K4me3 marker at the locus encoding myelin gene (He et al., 2007; Shen S. M. et al., 2008). Moreover, the decreased histone H4R3 symmetric methylation is followed by increased acetylation of H4K5 in OLs, and is rescued by pharmacological inhibition of HATs (Scaglione et al., 2018), which presents another good example that PRMT5 is critical for oligodendroglial differentiation by coupling histone methylation and acetylation. Taken together, the crosstalk between histone acetylation and methylation suggests the presence of a very complex enzymatic orchestra in OL, which coordinates to regulate the target genes transcription and govern OL growth and differentiation processes (Figure 1).

Another presence of crosstalk is that different post-translational modifications could occur at the same residue of histone proteins (Thomas, 2017b). Some lysine residues on histone 3 (e.g., H3K9 and H3K27) can be either acetylated or methylated, which may guide oligodendroglial development in different directions. Generally, histone acetylation at these sites is the open chromatin mark of oligodendroglial differentiation regulators, which secures OPCs within its mitosis (Koreman et al., 2018b). However, the methylation of the same sites exhibits multiple various effects on oligodendroglial development. For example, H3K9me3 could silence neuronal identity and membrane excitability gene expression and further facilitate oligodendroglial differentiation (Liu et al., 2015). Besides, the repressive role of H3K27me3 is selectively used by OPCs to silence different gene categories during its transition into myelinating OLs (Liu et al., 2015). Moreover, H3K27me3 could also promote OPCs cell fate choice from neural progenitor cells and stimulates OPCs proliferation (Sher et al., 2008; Koreman et al., 2018a). Therefore, the different histone modifications at the same site could induce various functional outcomes in OLs.

### Histone Acetylation and Methylation Alterations May Induce a Deleterious Epigenetic Memory in Oligodendrocyte

The concept of an epigenetic “memory” stably regulating the target gene expression is well-characterized in the study of imprinting and inheritance of parental traits (Heard et al., 2001; Zhou et al., 2005; Bantignies and Cavalli, 2006). In OL, epigenetic memory is established in two steps, characterized by deacetylation of histone lysine residues followed by the more stable methylation modifications (Shen et al., 2005), which depend on the enzymatic activities responsible for the chromatin changes of the OL lineage cells (Shen S. et al., 2008). This memory is responsible for the down-regulation and stable repression of inhibitory molecules in differentiated OLs (Marin-Husstege et al., 2006). Since histone acetylation and methylation enzymes activity are robustly dysregulated in schizophrenia (Peter and Akbarian, 2011; Qiu et al., 2017), they may disturb the epigenetic memory in OLs during the pathogenesis, and further lead to the heterochronic expression of transcriptional inhibitors in mature OLs. Importantly, the re-activation of inhibitors expression is not a random phenomenon, which indeed is selective for those genes whose levels progressively decreased during development or disease pathogenesis, due to the establishment of the “epigenetic memory” (Shen S. et al., 2008; Figure 1). Notably, environmental risk factors indicate the “scars” of early experience emerging across development by forming epigenetic maps, which may increase the sensitivity to future stress (Feinberg and Irizarry, 2010; Peña et al., 2019). Therefore, we deduce that environmental insults that interfere with the levels and activity of the histone modifying enzymes could results in a detrimental “epigenetic memory” in OLs, which may increase OLs and myelin susceptibility.

Antipsychotics, which are central to the treatment of schizophrenia (Kaar et al., 2020), have been noticed to regulate the histone acetylation and methylation at susceptibility genes (Swathy and Banerjee, 2017). Quetiapine is an atypical antipsychotic drug that has been proved to promote the differentiation and remyelination of OPCs (Xiao et al., 2008; Zhang et al., 2012). Recently, our study showed that quetiapine could significantly induce higher levels of histone methylation (H3K9me3) in myelinating OLs (Chen et al., 2019). Similarly, clemastine can also reverse the aberrantly decreased histone methylation in OLs, thereafter rescue the myelin deficits of prefrontal cortex as well as behavioral alterations (Liu et al., 2016). In addition, long-term use of second-generation antipsychotics can selectively up-regulate the expression of HDACs in human and mouse prefrontal cortex (Kurita et al., 2012). These results present good examples of applying antipsychotics to correct the adverse “epigenetic memory” in OLs during the pathogenesis, although the specificity of drug treatment on residues or enzyme orchestra still need further dissection.

## Conclusion

Cumulating evidence shows that environmental triggers could affect OLs and myelin deficits, probably through epigenetic mechanisms. As a major part of dysregulated histone modifications in schizophrenia, histone acetylation and methylation could affect oligodendroglial development, but in different ways. This mini-review provides a new perspective supporting transient histone acetylation in stage-specific ways, as well as stable histone methylation in a multidirectional manner in OLs. Furthermore, it highlights a coordination between these two modifications at several aspects, including the enzymatic orchestra and key sites specificities, which may form a detrimental epigenetic memory in OLs and increase the cellular susceptibility (Figure 1). However, several issues may need further dissection in the future study. (1) Which are the key histone modification sites that exhibit crucial functional outcomes in OLs? (2) Whether and how could the histone modification enzymes coordinate to secure the balance/priority of histone acetylation and methylation at these key sites? (3) How to achieve the specificity of drug treatment at the key sites with abnormal modification statuses? Overall, this work raises an intriguing possibility that precisely understanding the complexity of molecular orchestra of histone acetylation and methylation could be beneficial for developing novel treatment targets.

## Author Contributions

ML and XC designed this study and drafted the manuscript. All authors contributed to reviewing and editing the final manuscript.

## Conflict of Interest

The authors declare that the research was conducted in the absence of any commercial or financial relationships that could be construed as a potential conflict of interest.

## Publisher’s Note

All claims expressed in this article are solely those of the authors and do not necessarily represent those of their affiliated organizations, or those of the publisher, the editors and the reviewers. Any product that may be evaluated in this article, or claim that may be made by its manufacturer, is not guaranteed or endorsed by the publisher.
